# Evaluation of Unfavorable Cardiovascular and Metabolic Risk Factors in Children and Young Adults with Haemophilia

**DOI:** 10.4274/jcrpe.galenos.2018.2018.0292

**Published:** 2019-05-28

**Authors:** Melek Yıldız, Nihal Özdemir, Hasan Önal, Başak Koç, Beyza Eliuz Tipici, Bülent Zülfikar

**Affiliations:** 1 İstanbul University Cerrahpaşa Faculty of Medicine, Department of Pediatrics, İstanbul, Turkey; 2 İstanbul University Cerrahpaşa Faculty of Medicine and Oncology Institute, Department of Pediatric Hematology and Oncology, İstanbul, Turkey; 3 İstanbul University Cerrahpaşa Faculty of Medicine, Department of Pediatric Metabolism and Nutrition, İstanbul, Turkey; 4 İstanbul University İstanbul Faculty of Medicine, Department of Nutrition and Dietetics, İstanbul, Turkey

**Keywords:** Haemophilia, obesity, hypertension, metabolic syndrome

## Abstract

**Objective:**

Increased risk of unfavorable cardiovascular risk factors has been recognised in ageing patients with haemophilia (PwH), but needs further studies in younger patients. The purpose of this study was to assess obesity, hypertension (HT), metabolic variables, insulin resistance and metabolic syndrome in young PwH.

**Methods:**

Forty-eight haemophilia A and B patients and 35 age and sex matched healthy controls were included in the study. Anthropometric measurements, blood pressure (BP), fasting glucose and insulin levels, serum lipids and diet were evaluated. The metabolic syndrome was defined according to the criteria of the International Diabetes Federation for pediatric and adult age groups.

**Results:**

The mean age of PwH was 21±9 years (range, 6-40 years). Of those ≥18 years, 46% were were obese/overweight while there were no obese/overweight cases in the <18 year-old patients. Obesity was more prevalent in PwH with arthropathy (p=0.017). Seven percent of the PwH between 10 and 18 years-old and 25% of those ≥18 years had metabolic syndrome. There was no difference in metabolic syndrome frequency between PwH and controls >10 years-old (19.5% vs 10% respectively, p=0.34). Fifty percent of the PwH ≥18 years-old had elevated BP or HT. Fasting blood glucose levels of PwH were significantly higher compared to controls (p=0.02).

**Conclusion:**

Our study showed that obesity, HT and metabolic syndrome are frequent problems, especially in PwH with arthropathy. Early prevention and management of overweight, obesity and their sequelae must be addressed in clinical practice in order to maximize the overall health of the haemophilia population.

**What is already known on this topic?**

Patients with haemophilia were reported to have a reduced cardiovascular mortality due to a protective effect of having lifelong deficiency of factor 8 or 9. However, there is increasing evidence that this condition does not appear to be preventive.

**What this study adds?**

Cardiovascular and metabolic risk factors like overweight/obesity, elevated blood pressure/hypertension, prediabetes/diabetes and dyslipidaemia can be detected from early ages in patients with haemophilia.

## Introduction

In recent years, cardiovascular and metabolic risk has been defined in the ageing population of haemophiliacs. Studies in this area in young patients with haemophilia (PwH) are scarce (1,2). With increasing life expectancy of heamophilia patients, mortality and risk determination due to cardiovascular diseases has become an issue. While cardiovascular mortality was reported to be reduced in PwH due to a protective effect of having lifelong deficiency of factor 8 or 9 (3,4), there is an increasing evidence that this condition does not appear to prevent cardiovascular disease (5). Furthermore, Sun et al (2) recently demonstrated a significantly inferior microvascular endothelial function in haemophilia patients compared to healthy controls. Besides atherosclerosis, other established risk factors including obesity, hypertension (HT), dyslipidaemia, diabetes mellitus (DM) and family history for cardiovascular diseases are also known to play a crucial role in mortality and morbidity of these patients (1). Recently, Limjoco and Thornburg (6) studied these risk factors in a young haemophiliac population, aged 5-20 years, and identified modifiable risk factors for cardiovascular diseases.

Severe obesity in children and young adults is known to be associated with an increased prevalence of cardiometabolic risk factors, particularly among boys and young men (7). Haemophilia is characterized by progressive arthropathy, functional impairment and chronic joint pain. These are all barriers towards engagement in physical activity and may limit an individual’s ability to maintain a healthy weight (8,9). Therefore, obesity is a health issue in PwH as well as an aggravating factor for cardiometabolic and joint health (9).

Prevalence of HT in adults with moderate to severe haemophilia has been shown to be increased (10). Alperstein et al (11) reported an increased prevalence of HT in a hospitalized pediatric hemophilia population compared to a pediatric healthy male population, although this was not statistically significant (1.52% vs. 1.22%, p=0.26). Recently, Limjoco and Thornburg (6) reported high rates of overweight and obesity, (pre) HT and abnormal lipid levels in children and young adults with haemophilia.

The primary aim of this study was to assess obesity, HT, metabolic variables, insulin resistance and metabolic syndrome in children and young adults with haemophilia. We hypothesized that increased risk for cardiometabolic diseases could start from younger ages in PwH.

## Methods

### Study Design

This cross-sectional study was conducted in the Department of Pediatric Hematology and Oncology of Cerrahpaşa Medical Faculty and Oncology Institute of İstanbul University, between February 2010 and November 2010. Forty-eight PwH and 35 age and sex matched healthy controls were included. The study was approved by İstanbul Clinical Research Ethics Committee No:1 (No: C-009/2010). Informed consent was obtained from parents for age groups 6-12 years, from both subjects and parents for age groups 12-18 years, and from subjects only in those older than 18 years, in accordance with the Declaration of Helsinki.

### Patients

During the regular outpatient clinic visit for hemophiliac patients, consecutive patients were asked to participate in the study. Forty-eight male patients, aged 6-40 years, with hemophilia A and B were included in the study irrespective of the severity of their disease. Patients with a coagulation factor level of less than 1% of normal were classified as severe, 1-5% of normal were classified as moderate, and 5-40% of normal were classified as mild (12). The past medical records of the participants were examined. Age of diagnosis, annual spontaneous or traumatic bleeding rate, annual factor consumption, presence of chronic arthropathy, whether physiotherapy or home exercise was undertaken, sports and dietary habits, presence of other systemic diseases and/or other complications of haemophilia, such as carrier status of HBV, HCV, HIV or presence of inhibitor were recorded. Collected data items also included self-reported family history for DM, HT, coronary artery disease (CAD) (male <55 years, female <65 years) and dyslipidaemia. The annual factor consumption per kilogram of body weight (in international units/kg) was determined for each patient for the 12-month period prior to enrollment. Factors used for elective interventional and surgical procedures were not taken into account. Patients with a history of known cardiovascular disease were not included in the study. Blood samples were taken as part of the clinical follow-up of patients.

### Control Group

The control group consisted of 35 age-matched, random male subjects, with no history of congenital or acquired bleeding disorder, cardiovascular disease or chronic disease, who presented to the outpatient clinics of the pediatric or internal medicine departments. Age, sports and dietary habits, self-reported family history for DM, HT, CAD (male <55 years, female <65 years) and dyslipidaemia were recorded.

**Assessment of exercise and nutritional status:** In the study and control groups, for individuals under 18 years, at least three days and 30 minutes regular exercise a week; for individuals aged 18 years and older, at least two days and 30 minutes a week were classified as those who perform regular exercise and others were classified as non-performing.

Dietary intake was evaluated by an experienced dietitian using a 3-day food record. Subjects were given detailed oral and written instructions regarding the completion of a 3-day food record, consisting of two midweek days and one weekend day. In order to determine the amounts of consumed foods correctly, information was given about measuring cups such as water glass, tea glass, teaspoon, tablespoon, serving spoon and bowl. Energy and nutrient intake was analyzed by a computerized food analysis program adapted for our country (BeBis4 software program, Turkish version, Stuttgart, Germany) and evaluated according to the recommendations of the Turkish Dietary Guidelines (13). A percentage level of sixty-six or less of the references was used as the criterium for inadequate nutritional intake (14). Over 300 mg daily cholesterol intake was considered as high intake.

**Anthropometric measurements:** All participants underwent a complete physical examination, including standardized measurement of weight, height and waist circumference, in duplicate. Body mass index (BMI) standard deviation (SD) scores values were calculated for children <18 years, using Turkish national reference data (15). Subjects with BMI ≥95% were defined as obese and with BMI ≥85% and <95% as overweight. BMI was calculated for adults and classified as underweight, normal weight, overweight or obese based on World Health Organization classification (16). Waist circumference was evaluated according to NHANES 3 reference limits appropriate for age and sex (17). Blood pressure (BP) was evaluated by three consecutive measurements. In children <13 years of age, HT was defined as systolic or diastolic BP ≥95^th^ percentile according to the recent sex, age and height tables (18). For adolescents ≥13 years and adults, HT was defined as systolic and/or diastolic BP ≥140/90 mmHg and elevated BP was defined as systolic and/or diastolic BP ≥120/80 mmHg according to 2017 guidelines (18,19).

**Biochemical assessment:** In all subjects, fasting glucose, total cholesterol, low-density lipoprotein-cholesterol, high-density lipoprotein-cholesterol, triglyceride, uric acid (measured by enzymatic colorimetric method using Abbott Architect c8000 autoanalyzer) and insulin (measured with chemiluminescence, by Abbott Architect i2000) levels were evaluated. Hyperglycemia was defined as a fasting glucose level of ≥100 mg/dL. Insulin resistance was estimated by the Homeostasis Model Assessment of Insulin Resistance (HOMA-IR) formula, that is fasting serum insulin (µU/mL) x fasting plasma glucose (mmol/L)/22.5, as described by Matthews et al (20). For adults, a HOMA-IR value above 2.7 was considered as insulin resistance (21). For children and adolescents, insulin resistance was evaluated by HOMA-IR values higher than ≥97th percentile for age and sex (22).

The criteria of the International Diabetes Federation, in children above 10 years (23) and adults (24) were used for assessment of metabolic syndrome.

### Statistical Analysis

The data were analyzed using the Statistical Package for Social Sciences program, version 18.0 (IBM Inc., Chicago, IL, USA). For baseline characteristics, a descriptive statistical analysis was performed using percentages for categorical variables, mean ± SD for normally distributed continuous variables and median (range) for skewed continuous variables. Differences between two groups were tested using two-sample t-test or Mann-Whitney U test for continuous variables and chi-square test or Fisher’s exact test for categorical variables, as appropriate. Results were evaluated at 95% confidence interval and a p value less than 0.05 was considered statistically significant.

## Results

A total of 48 haemophilia patients and 35 age and sex matched healthy controls were included in the analysis. The demographic characteristics between the groups were similar. Mean ages of the haemophilia and control groups were 20.5±9.1 years and 21.4±9.0 years, respectively (p=0.65). There was no difference in weight, height, BMI and waist circumference values of PwH and controls (Table 1). Median age at diagnosis was 11 (1-129) months among haemophilia patients. Sixty-six percent of haemophilia A and 50% of haemophilia B patients were on prophylaxis, others were on demand therapy. Median annual factor consumption for haemophilia A and B patients were 1846 IU/kg (0-3600) and 840 IU/kg (318-2434), respectively. The frequency of PwH with inhibitors was 8.3% and these subjects were mainly severe type haemophilia A cases. Arthropathy was present in 61.9% of the haemophilia A and 66.7% of haemophilia B patients; and mainly in patients of severe type over 18 years old. Only a few of the patients were getting physiotherapy (5% of haemophilia A and none of the haemophilia B patients).

Energy, protein, fat, carbohydrate, fiber and cholesterol intakes by age and sex were calculated in the haemophilia and control groups according to their food consumption records (Table 1). Energy intake was significantly higher in the control group (p=0.02), but other nutrient intakes were similar. Subjects in both study and control groups were exposed to similar amounts of regular exercise. Haemophilia patients of normal weight were taking less regular exercise compared to those with overweight or obesity, 14.3% of the normal weight group compared to 23.1% of the overweight/obese patients although this was not significant (p=0.66).

Biochemical assessment of all participants is shown in Table 2. Fasting blood glucose levels of PwH were higher compared to controls (p=0.02). However, serum triglyceride concentrations were significantly lower in the haemophilia group (p=0.008). Total cholesterol levels were somewhat lower in the haemophilia group, but the difference did not reach statistical significance. Forty-six percent of PwH over 18 years were overweight/obese, however none of the patients younger than 18 years old were overweight/obese. Obesity was more prevalent in PwH with arthropathy (p=0.017). When metabolic syndrome was assessed in different age groups, none of the patients <10 years old had metabolic syndrome, 7.7% of patients between 10 and 18 years old and 25% of PwH between 18 and 40 years old had metabolic syndrome. Fifty percent of PwH >18 years had elevated BP/HT vs 23% of those ≤18 years (p=0.03). The frequency of elevated BP/HT remained higher in all subjects in the haemophilia cohort, although not statistically significant, when compared to controls (35.5% vs. 28.6%, p=0.51). When PwH and controls over 10 years were compared for metabolic syndrome, no statistically significant difference was found (19.5% and 10% respectively, p=0.34). Comparison of metabolic variables among haemophilia patients and controls are shown in Table 3. In addition, the Spearman correlation analysis did not show any correlation between annual factor consumption and any of the metabolic parameters in PwH.

## Discussion

Our study showed that obesity, HT and metabolic syndrome are frequent problems in PwH, especially in those over 18 years with arthropathy. Early prevention and management of overweight, obesity and their sequelae must be addressed in clinical practice in order to maximize the overall health of the haemophilia population. Therefore, assessment of cardiovascular and metabolic risk factors, beginning from early childhood, is crucial for this specific patient population.

The relationship between haemophilia and cardiovascular risk is not yet well understood (25). In several cohort studies Haemophilia has traditionally been regarded as a protective state for thrombosis due to hypocoagulability (3,4). However, some studies indicated a potential negative effect of haemophilia (26), while some found no substantial effect (27). As obesity is an important risk factor for cardiovascular disease, the impact of obesity in the haemophilia population on cardiovascular disease has been reviewed (9). These authors recommended implementing general guidelines for weight management in the context of the haemophilia care team.

Overall life expectancy and quality of life among the haemophiliac population have increased in recent years, primarily because of the reduction in mortality/morbidity due to infections and advances in factor replacement therapy. However, older patients who had been treated with on demand therapy still have a variety of orthopedic problems. In our study, overweight and obesity were frequent in subjects over 18 years with target joints. This finding may be attributed to reduced engagement in physical activity to prevent bleeding and to protect the joints. Besides, target joints progressively lead to pain, restriction of movement and potentially irreversible structural damage, the hallmarks of haemophilic arthropathy. The associated reduced physical activity results in weight gain. Furthermore, the reduced mobility and loss of muscle function leads to muscle atrophy, which may in turn increase the risk of weight gain (28). Furthermore, in a Dutch haemophilia cohort, it has been reported that overweight/obesity itself increased the number of joint bleeds and reduced function in the lower limbs (29). Recently, Limjoco and Thornburg (6) reported high rates of overweight and obesity in a relatively younger haemophilia cohort (mean age 12 years, range 5-20 years). However, these authors identified no difference in target joints based on weight category (30% in normal weight vs. 25% in overweight or obese, p=0.74). They suggested that the impact of overweight and obesity on joint disease may have been offset by the high rate of prophylaxis or that it may manifest over longer periods of time in follow-up.

Another outcome of increase in life expectancy of PwH is experiencing cardiovascular complications. HT is one of the most relevant cardiovascular risk factors that has gained attention, since it is also a major risk factor for intracranial hemorrhage in PwH (30). There are some studies documenting an increased rate of HT in adults with hemophilia (10,31) but little is currently known about its prevalence and severity. Increased prevalence of HT may be the result of the regular visits of these patients to clinics and getting the diagnosis of HT or due to intraparenchymal hemorrhages in the kidneys (32). Recently, a slightly increased prevalence of HT was reported in a pediatric hemophilia population, thus raising awareness of the need for assessment of BP even in young PwH (11). Our study showed that the prevalence of elevated BP and HT was higher, especially in PwH over 18 years, although this finding was not statistically significant. Nevertheless, the clinical difference noted in the hemophiliac group demonstrates a trend and warrants further study. BP measurements should be a part of standard care in PwH early in their life, with the possible consideration of early intervention.

Although none of the patients had DM in our cohort, higher concentrations of fasting blood glucose, which predicts diabetes, were observed. The same subpopulation was affected by both increased blood glucose levels and obesity, as expected. Biere-Rafi et al (1) identified a higher proprotion of PwH with hyperglycemia than controls. In contrast Alperstein et al (11) reported a low prevalence of DM in a pediatric haemophilia population. While the prevalence of dyslipidaemia was similar among haemophiliacs and the control group, mean serum concentrations of triglyceride were significantly lower in our haemophilia group. Additional research is required to determine whether blood glucose and lipid screening should start earlier for children with haemophilia.

The frequency of metabolic syndrome in our pediatric PwH (aged 10-18 years) was higher than Turkish data on healthy schoolchildren, aged 10-19 years, according to the criteria of IDF (7.7% vs 2.3%, respectively) (33). However, for the adult age group of PwH, frequency of metabolic syndrome was comparable with previously reported data (25% in PwH vs. 31.2% in Turkish adult males) (34).

### Study Limitations

This study has several limitations. Our haemophilia patients were very heterogeneous with a wide age range (child, adolescent and adult), with both haemophilia A and B and all degrees of severity. Therefore, most of the subgroup analyses could not be performed and the relationship between cardiometabolic risk factors and severity of disease could not be analyzed. Further studies should include a more homogenous study population. Furthermore, our data were collected from past medical records and at only one outpatient clinic visit rather than over time. Repeated BP measurements on different days are needed for the accurate estimation and diagnosis of HT, and the exclusion of “white coat” HT. Although we were not able to collect measurements on different days, we referred all subjects with at least one episode of elevated BP/HT for further assessment. Another limitation of our study was that we did not have information about smoking and alcohol use, which will have an impact on cardiovascular and metabolic parameters. Furthermore, we asked about the subject’s routine exercise status, but did not question them about how many hours they spend watching TV, playing computer games and using mobile phones, which are risk factors for the development of obesity. Further studies should be designed to follow-up patients longitudinally.

## Conclusion

In conclusion, cardiovascular and metabolic risk factors like overweight/obesity, elevated BP/HT, prediabetes/DM and dyslipidaemia can be detected from very early ages in PwH. Screening from early ages for cardiovascular risk factors and considering early intervention and management might help to improve the general health status of this specific patient group and reduce morbidity.

## Figures and Tables

**Table 1:**
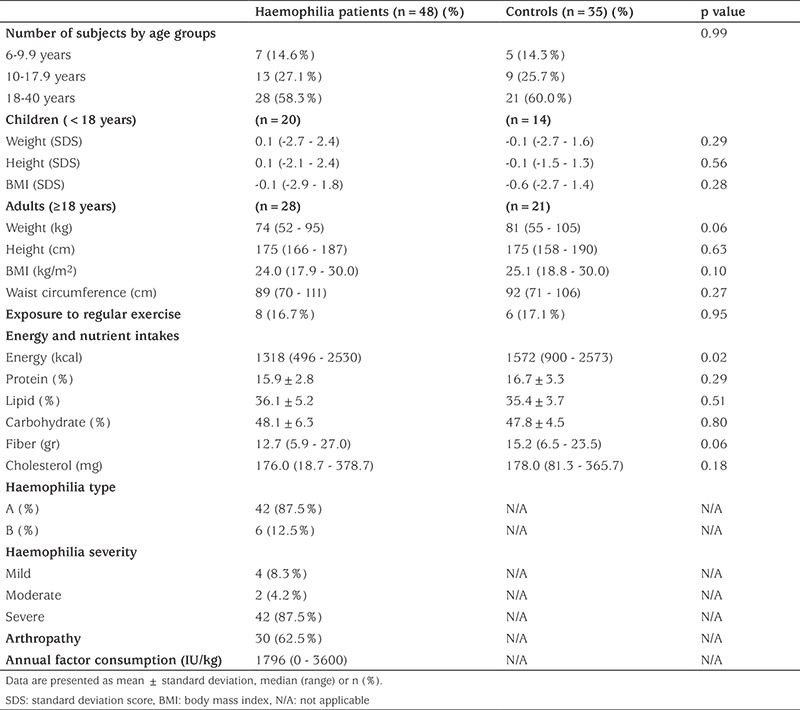
Clinical and anthropometric data in patients with haemophilia and in healthy controls

**Table 2:**
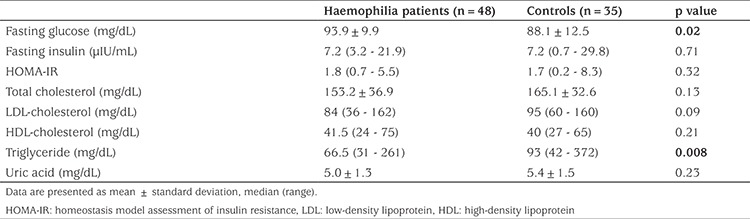
Biochemical profile of patients with haemophilia and healthy controls

**Table 3:**
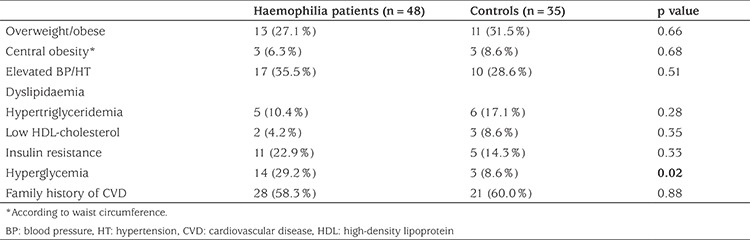
Frequency of metabolic syndrome components
